# A method of “Noninjecting Resection using Bipolar Soft coagulation mode; NIRBS” for superficial non-ampullary duodenal epithelial tumor: a pilot study

**DOI:** 10.1186/s12876-024-03439-w

**Published:** 2024-10-01

**Authors:** Mitsuo Tokuhara, Yasushi Sano, Yoshifumi Watanabe, Hidetoshi Nakata, Hiroko Nakahira, Shingo Furukawa, Takuya Ohtsu, Naohiro Nakamura, Takashi Ito, Ikuko Torii, Takeshi Yamashina, Masaaki Shimatani, Makoto Naganuma

**Affiliations:** 1 Department of Gastroenterology, Japan Community Healthcare Organization Hoshigaoka Medical Center, 4-8-1 Hoshigaoka, Hirakata, Osaka 573-8511 Japan; 2 Gastrointestinal Center, Sano Hospital, Kobe, Hyogo Japan; 3 Third Department of Internal Medicine, Division of Gastroenterology and Hepatology, Kansai Medical University Hirakata Hospital, Hirakata, Osaka Japan; 4 Department of Pathology, Japan Community Healthcare Organization Hoshigaoka Medical Center, Hirakata, Osaka Japan

**Keywords:** Bipolar snare, Noninjecting, Soft coagulation mode, Superficial nonampullary duodenal epithelial tumor, SNADET, Duodenal lesion

## Abstract

**Background:**

Complete endoscopic resection of superficial non-ampullary duodenal epithelial tumors (SNADETs) is technically difficult, especially with an extremely high risk of adverse event (AE), although various endoscopic resection methods including endoscopic mucosal resection (EMR), underwater EMR (UEMR), and endoscopic submucosal dissection (ESD) have been tried for SNADETs. Accordingly, a novel simple resection method that can completely resect tumors with a low risk of AEs should be developed.

**Aims:**

A resection method of Noninjecting Resection using Bipolar Soft coagulation mode (NIRBS) which has been reported to be effective and safe for colorectal lesions is adapted for SNADETs. In this study we evaluated its effectiveness, safety, and simplicity for SNADETs measuring ≤ 20 mm.

**Results:**

This study included 13 patients with resected lesions with a mean size of 7.8 (range: 3–15) mm. The pathological distributions of the lesions were as follows: adenomas, 77% (*n* = 10) and benign and non-adenomatous lesions, 23% (*n* = 3). The *en bloc* and R0 resection rate was 100% (*n* = 13). The median procedure duration was 68 s (32–105). None of the patients presented with major AEs including bleeding and perforation.

**Conclusions:**

Large studies such as prospective, randomized, and controlled trials should be conducted for the purpose of validating effectiveness, safety, and simplicity of the NIRBS for SNADETs measuring ≤ 20 mm suggested in this study.

## Introduction

Superficial non-ampullary duodenal epithelial tumors (SNADETs) were previously considered rare diseases [1–3]. However, in recent years, advances in endoscopic techniques and increased awareness of this disease by endoscopists have led to increased opportunities to detect SNADETs [4, 5]. Pancreaticoduodenectomy (Whipple’s procedure) is the standard treatment for duodenal cancer. However, the substantial morbidity and mortality rates of this procedure are 30%–40% and 1%–4%, respectively [6–9]. Moreover, it is extremely invasive for SNADETs [10]. Endoscopic resection (ER) can preserve the organ and thus maintain postoperative quality of life [11]. However, ER in the duodenum is more difficult and potentially risky than in the other digestive tract due to physical factors. The duodenum is heavily vascularized, and the scope’s inadequate capacity to maneuver in this tight space hinders a proper approach to the lesion [12]. In addition, the duodenal wall is extremely thin and can be easily perforated. Three main methods (cold snare polypectomy [CSP], endoscopic mucosal resection [EMR] and endoscopic submucosal dissection [ESD]) are used for ER of the duodenum [13–22]. Although current guidelines suggest CSP for small (< 6 mm) duodenal lesions [23], the adaptation of CSP should be cautious for large lesions or cancerous lesions because it may result in incomplete mucosal layer resection [24–26]. In a large prospective trial on EMR for SNADETs (118 duodenal lesions, mean size 15 mm) [27], authors reported that EMR has an excellent complete resection rate (94.1%), but the adverse event (AE) rate was 22.9% (delayed bleeding; 18.6%, perforation; 4.2%). It suggested that these consequences were linked to the damage brought on by electrocautery performed for large SNADETs [12]. Although the ESD can be expected to accomplish *en bloc* resection even for extensive duodenal lesions, duodenal ESD is extremely challenging to perform and AE-rate of ESD in the duodenum is higher than that in other organs [28]. Delayed perforation occasionally requires emergent surgery. Thus, it is one of the most important AEs in the duodenum. In case of delayed perforation, patients may require highly invasive surgical interventions such as pancreaticoduodenectomy or prolonged hospitalization even if conservative treatment is possible [11]. Therefore, a novel resection method that can completely resect tumors with a low risk of perforation should be developed. Recently, an ER method called “Noninjecting Resection Using Bipolar Soft Coagulation Mode (NIRBS)” has been reported to be effective and safe for colorectal lesions [29]. This is a novel method in which the lesion is grasped, including the surrounding normal mucosa without injection into the submucosal layer, sufficiently squeezed, and resected with short-time energization using the bipolar snare set in the soft coagulation mode, which has a minimal burn effect. Hence, the current study aimed to evaluate the efficacy, safety, and simplicity of NIRBS in SNADETs.

## Methods

### Patients and target lesions

SNADETs detected via upper gastrointestinal endoscopy from September 2021 to May 2024 at Japan Community Healthcare Organization Hoshigaoka Medical Center, Osaka, Japan, were consecutively evaluated. The inclusion criteria were as follows: (1) patients endoscopically diagnosed with SNADETs (biopsy before treatment was not required); (2) those with lesions measuring ≤ 20 mm; (3) those with lesions located between the 1st and 3rd part of the duodenum, except those near the pyloric ring; (4) those aged ≥ 20 years; and (5) those who provided a written informed consent. The endoscopic diagnosis of SNADET was based on the white light imaging scoring system, as reported in a previous study [30]. Chromoendoscopy and narrow band imaging findings were also taken into consideration. The procedure was performed with the temporary discontinuation of antithrombotic therapy.

### Procedures

**Fig. 1:**
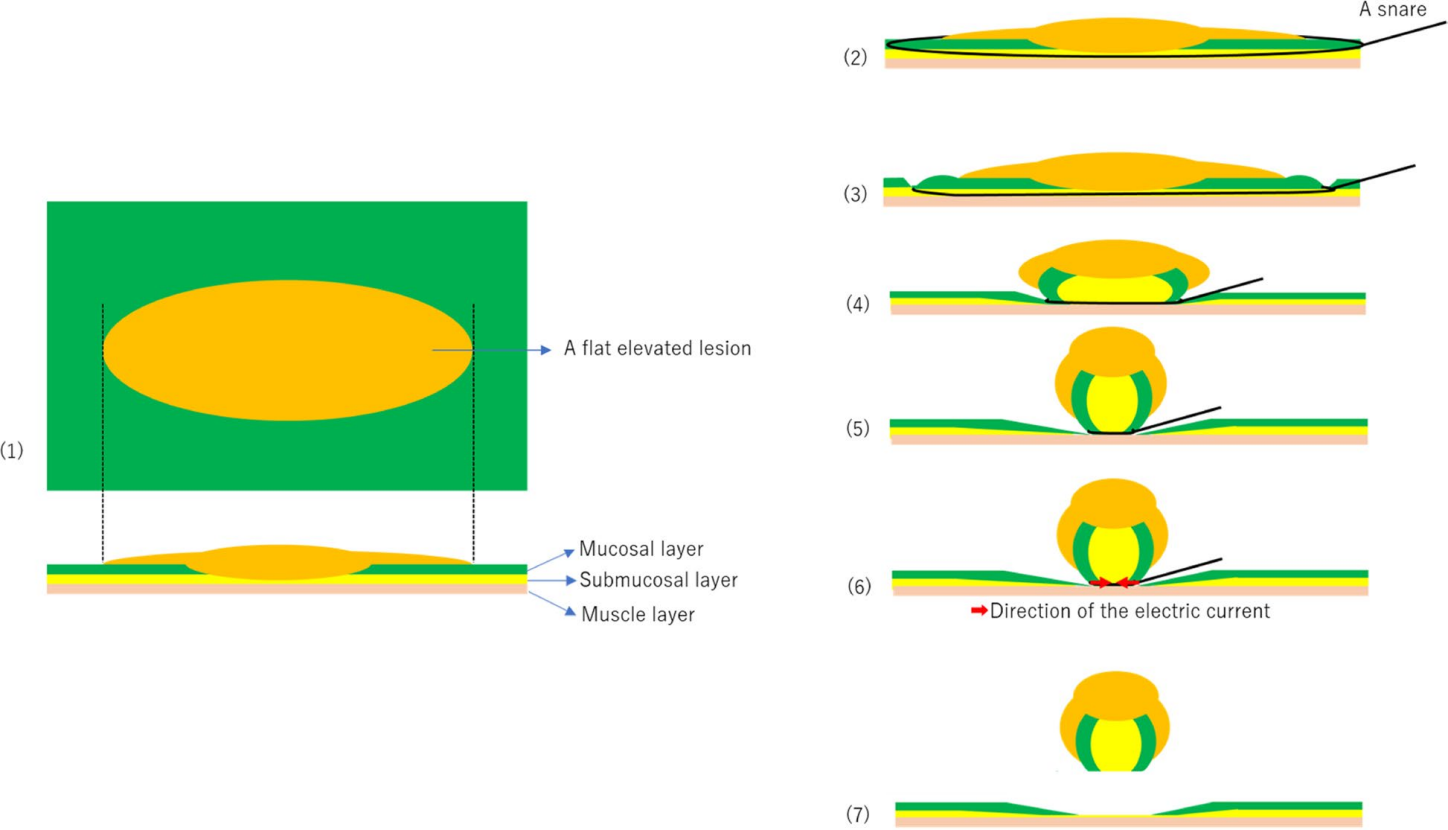
NIRBS procedure (Schema). (1); A flat elevated lesion. (2); Snaring. (3); Squeezed with sucking the air. (4), (5); The snare squeezes the lesion like sliding just above the muscle layer, and the softest submucosal layer is lifted upwards by the strangled mucosal layer due to squeezing with the quick juggling technique. The snare can avoid involving of the muscle layer due to the quick juggling. (6); Energized while continuing to squeeze with the quick juggling technique. (7); The submucosal deep layer just above the muscle layer is resected

**Fig. 2:**
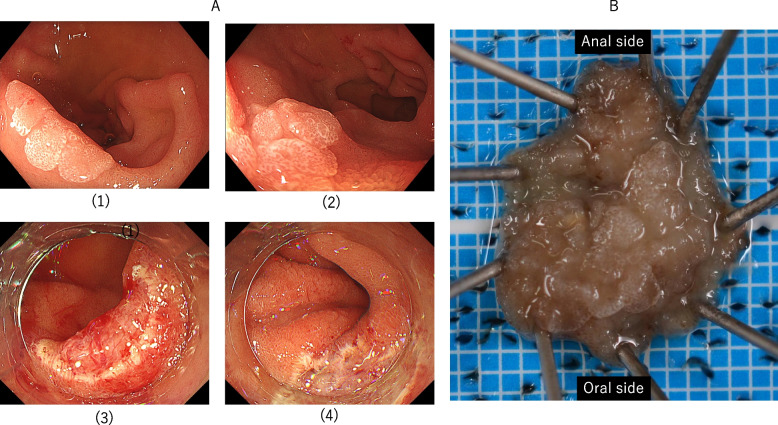
Superior duodenal angle, 0–IIa-type SNADETs. **A**: Endoscopic findings. (1) The lesion spreading to the fold. (2) The lesion spreading to the anal side. (3) Oral side view after NIRBS. The portion just above the muscle layer was resected. (4) Anal side view after NIRBS. **B** Macroscopic view of the resected specimen. The pathological findings were tubular adenoma intestinal type, pHM0, and pVM0

NIRBS was defined as conditions meeting (1) – (5) [29]. (1) The XEMEX Bipolar Snare S (width, 26 mm; length, 62 mm; Zeon Medical Inc., Tokyo, Japan) was used. (2) The electrosurgery generator mode was set to the soft coagulation mode at 30 W and Effect 5 (VIO 300D, ERBE Elektromedizin GmbH Co., Ltd., Tubingen, Germany) or the soft coagulation mode at Effect 3.3 (VIO 3, ERBE Elektromedizin GmbH Co., Ltd., Tubingen, Germany). (3) Injection into the submucosal layer was not performed. (4) The lesion, including the surrounding normal mucosa, was grasped extensively due to snaring with sucking the air. (5) With continuous sufficient squeezing using the quick juggling technique [29], each lesion was resected according to its respective energization, which was as follows: within 2, 5, and 10 s for those measuring ≤ 5, 6–10, and > 10 mm, respectively (Figs. 1 and 2). The quick juggling technique was continuously applied from energization to resection. The procedure was performed using GIF-H290T (Olympus Medical Systems Co., Ltd., Tokyo, Japan). After resection, clipping disclosure was performed using long clips (Olympus Medical Systems Co., Ltd.).

### Study design

This single-center, retrospective study evaluated the use of NIRBSs for SNADETs. The primary endpoint was *en bloc* resection rate. The secondary endpoints were rate of AEs (perforation and bleeding) including delayed AEs and mean procedure duration.

### Adverse events

Delayed bleeding was defined as clinical evidence of bleeding after the procedure, evidenced by hematemesis or melena requiring endoscopic treatment. Delayed perforation was defined as the presence of perforation not detected during and just after procedural completion, free air on radiography or computed tomography scan after the procedure, and perforation at the resection site on subsequent endoscopy. Delayed AEs were defined as AEs occurring within 1 month after the end of the procedure.

### Procedure duration

Procedure duration was defined as the time from snare insertion into the working channel of the scope to lesion resection.

## Results

**Fig. 3:**
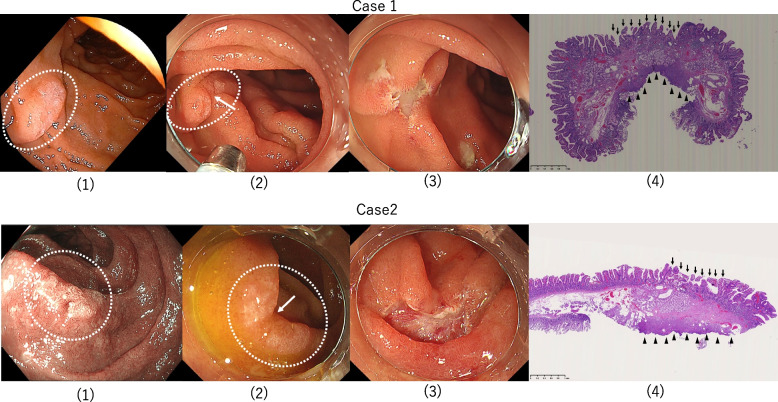
Duodenal second portion, 2 cases of 0–IIa-type SNADETs with a biopsy-induced scar. (1) Before biopsy. (2) After biopsy. The arrow ( →) indicates the biopsy-induced scar. (3) Resected portion after NIRBS. (4) Pathological findings using the resected specimen (low magnification). The arrows ( →) indicate the biopsy-induced scar. The arrowheads (►) indicate the cut end

Table 1 showed characteristics and procedure-related outcomes. The location distribution of the lesions was as follows: bulb, 23% (*n* = 3); second portion and preampulla, 54% (*n* = 7); and second portion and postampulla, 23% (*n* = 3). The morphological types of the lesions were as follows: sessile type, 38% (*n* = 5); superficial elevated type, 38% (*n* = 5); and superficial depressed type, 23% (*n* = 3). The median diameter size of the tumors was 7.8 (3–15) mm. The pathological distributions of the lesions were as follows: adenomas, 77% (*n* = 10) and benign and non-adenomatous lesions, 23% (*n* = 3). The *en bloc* and R0 resection rate was 100% (*n* = 13). Two patients presented with biopsied scar (Fig. 3). The mean distance between the deepest portion of the tumor and the vertical margin was 1638 μm (513–4224). The median procedure duration was 68 s (32–105). None of the patients presented with major AEs including bleeding and perforation.

**Table 1:** Characteristics and procedure-related outcomes

Patients	13 cases
Male	8(62%)
Female	5(38%)
Median age (range, years)	70(59–86)
Location	
Bulb	3(23%)
Second portion, preampulla	7(54%)
Second portion, postampulla	3(23%)
Morphology	
Sessile type	5(38%)
Superficial elevated type	5(38%)
Superficial depressed type	3(23%)
Median tumor diameter size (range, mm)	7.8(3–15)
Pathological type	
Adenoma	10(77%)
Intramucosal carcinoma	0(0%)
Benign and non-adenomatous lesion	3(23%)
En bloc and R0 resection	13/13(100%)
The mean distance between the deepest portion of the tumor and the vertical margin (range, μm)	1638(513–4224)
Median procedure duration (range, sec)	68(32–105)
Adverse events	
Intraprocedural or delayed perforation	0
Intraoperative bleeding	0
Delayed bleeding	0

## Discussion

ER such as EMR and ESD can preserve the organs and maintain quality of life. However, ER of the duodenum is technically more challenging to perform. Moreover, the risk of delayed AE in the duodenum is higher than that of other organs. This phenomenon is caused by various anatomical features such as a narrow lumen, a C-loop that reduces endoscope stability, the presence of the Brunner’s glands in the deep mucosal and submucosal layers in the bulb that stiffen the wall and lead to poor mucosal lifting, a thin deep muscle layer that increases the risk of perforation, and difficulties associated with accessing sites if emergency or salvage surgery is required [31–33]. Further, due to exposure to bile and pancreatic juice from the duodenal papillae, the risk of delayed AE such as bleeding and perforation is significantly higher in the duodenum than in other organs, even if the treatment is completed safely. In case of delayed perforation, a highly invasive surgery such as pancreaticoduodenectomy or prolonged hospitalization may be required even if conservative treatment is possible. Therefore, SNADETs require a resection method that is simple, can be used for *en bloc* resection, and have a low risk of AEs. CSP [22] is effective for small duodenal lesions. However, the adaptation of CSP should be cautious for large lesions or cancerous lesions because it may result in incomplete mucosal layer resection [24–26]. Conventional EMR, which requires submucosal injection before snaring, is effective in the duodenal second portion with less Brunner’s glands. However, in the duodenal bulb with rich Brunner’s glands, the adaptation of conventional EMR should be cautious because it may result in insufficient mucosal lifting. Duodenal ESD is extremely challenging to perform [28]. In a previous report [13], the mean procedure duration and rate of AE for conventional EMR and ESD were about 6 min and 3.6%, and about 88 min and 17.9%, NIRBS in this study tended to have lower mean procedure duration (about 1 min) and lower rate of AE (0%) than those of conventional EMR and ESD. Recently, some studies have utilized modified EMR techniques including underwater endoscopic mucosal resection (UEMR) [34–38] and gel immersion endoscopic mucosal resection (GIEMR) [39], which have attracted attention due to their safety and efficacy in SNADETs. The mean procedure duration of NIRBS (68 s) in this study had a shorter procedure duration than UEMR and GIEMR (600 and 300 s) [39]. This is because NIRBS is a simple method which special techniques were not required, and only resection by wide snaring, squeezing, and energization was conducted. However, UEMRs or GIEMRs are considered to be difficult to be easily submerged for all SNADETs in the duodenum with poor maneuverability. A Japanese study performed in 2023 first reported the safety and efficacy of NIRBS in 746 colorectal lesions [29]. This is the first report on the use of NIRBS for SNADETs. Bipolar snares generally cause less tissue damage than monopolar snares [40–43]. The significant difference between the methods of previous reports using bipolar snare and NIRBS is that the latter is set in the soft coagulation mode, thereby causing less tissue damage than the forced coagulation mode [29]. The soft coagulation mode causes minimal carbonization and burning because the voltage is controlled and does not generate electrical sparks [44, 45]. The bipolar snare, which is flexible, can slide into the loosest submucosal layer between the mucosal and muscle layers even if the mucosa is widely grasped and squeezed [29]. The mucosal and muscle layers were consistently separated via sufficient squeezing with the quick juggling technique, then, the submucosal layer was completely resected with the soft coagulation having a minimal burn effect. The snare can avoid involving of the muscle layer due to the quick juggling. The submucosal deep layer can be resected by sliding just above the muscle layer without perforation in the thin-walled duodenum. However, we should be very careful not to damage the muscle layer because of having a minimal burn effect, even though it is bipolar soft coagulation mode. Although we used this large size snare (width, 26 mm; length, 62 mm) in this study for the purpose of accomplishing *en bloc* resection for even lesions of nearly 20 mm, it may be better to use smaller size snares for small lesions in order to reduce the resection of the normal portion. In this study, the *en bloc* resection rate was 100% (*n* = 13), and the distance between the deepest portion of the tumor and the vertical margin in all lesions was sufficient. Furthermore, *en bloc* resection could be performed easily even in the two patients with biopsied scars, although ER can be more challenging to perform in patients with biopsied scars [46]. None of the patients in this study developed AEs such perforations and bleedings. Therefore, NIRBS may be suitable for the ER of SNADETs.

The current study had several limitations. For example, it was retrospective in nature and was performed at a single facility. This study is insufficiently to suggest that the method of NIRBS has great benefits compared to more widely accepted endoscopic resection methods such as CSP, EMR, and ESD, because this study was a first pilot study of NIRBS for SNADETs, and only a small number of cases were included. If *en bloc* resection with NIRBS for SNADETs can be performed effectively, safely, and simply without perforation, the benefits will be immeasurable. However, unless larger studies than this study are underwent, duodenal NIRBS is considered to be only an alternative to the existing methods. Hence, future multicenter, prospective, randomized, controlled trials should be conducted to evaluate and standardize the use of NIRBS for SNADETs.

## Data Availability

All data generated or analyzed during this study are included in this published article.

## Abbreviations

SNADETs: Superficial nonampullary duodenal epithelial tumors
NIRBS: Noninjecting Resection using Bipolar Soft coagulation mode
CSP: Cold snare polypectomy
ESD: Endoscopic submucosal dissection
EMR: Endoscopic mucosal resection
ER: Endoscopic resection
AE: Adverse event
UEMR: Underwater endoscopic mucosal resection
GIEMR: Gel immersion endoscopic mucosal resection
